# LODDS-based nomogram for breast cancer-specific survival in surgically treated breast cancer in young patients

**DOI:** 10.17305/bb.2026.14254

**Published:** 2026-06-24

**Authors:** Ziqiang Wang, Zhenlin Yang, Haojie Zhang

**Affiliations:** 1 Department of Breast Surgery, Shandong Medical and Pharmaceutical University Hospital, Binzhou, Shandong, China; 2 Shandong Medical and Pharmaceutical University, Binzhou, Shandong, China; 3 Department of Thyroid Surgery, Shandong Medical and Pharmaceutical University Hospital, Binzhou, Shandong, China

**Keywords:** Breast cancer, log odds of positive lymph nodes, LODDS, prognosis, nomogram, LASSO regression.

## Abstract

Traditional tumor staging relies on absolute lymph node counts, making it susceptible to stage migration, particularly in young breast cancer (YBC) patients with aggressive disease. This study assessed the prognostic value of the continuous log odds of positive lymph nodes (LODDS) for breast cancer-specific survival (BCSS) and developed a predictive nomogram utilizing data from 9,989 surgically treated YBC patients (aged 20–39) sourced from the Surveillance, Epidemiology, and End Results (SEER) database (2010–2015). A multivariable Cox proportional hazards model, adjusted to account for non-proportional hazards, established continuous LODDS as an independent risk factor for poorer BCSS (hazard ratio [HR] ═ 1.60, 95% confidence interval [CI]: 1.45–1.77, *P <* 0.001), without relying on data-driven dichotomization. We constructed a nomogram that integrates continuous LODDS with clinicopathological features selected via least absolute shrinkage and selection operator (LASSO). Under a 1,000-resample bootstrap optimism-correction, the nomogram exhibited strong discriminatory power (corrected concordance index [C-index] ═ 0.777), surpassing the traditional tumor-node-metastasis (TNM) staging model (C-index = 0.736). Moreover, the continuous LODDS-integrated model improved 5-year mortality risk reclassification (continuous net reclassification improvement [NRI] ═ 0.278, integrated discrimination improvement [IDI] ═ 0.034; both *P <* 0.001). The model also demonstrated high calibration accuracy (5-year Brier score = 0.0757) and presented a greater estimated net benefit in Decision Curve Analysis (DCA). In conclusion, continuous LODDS offers critical incremental prognostic information beyond traditional staging. The proposed nomogram serves as an internally validated prediction model for risk stratification in YBC patients.

## Introduction

Breast cancer is the most prevalent malignancy among women globally [1]. A worldwide trend indicates an increasing incidence of breast cancer in younger patient populations [2, 3]. Within this demographic, young breast cancer (YBC) patients, typically defined as those diagnosed at age 40 or younger [4], represent a distinct and challenging clinical subgroup. Compared to older patients, tumors in younger individuals generally exhibit more aggressive biological characteristics, such as higher histological grades and more complex molecular subtypes, an elevated recurrence risk, and poorer overall survival prognoses [5, 6]. Given their longer life expectancy and reduced tolerance for the toxicities associated with overtreatment, precise prognostic risk stratification is critical for YBC patients. Such stratification not only aids clinicians in identifying high-risk individuals but also forms a crucial foundation for personalized adjuvant treatment strategies.

Lymph node status has long been recognized as a pivotal prognostic determinant for early and locally advanced breast cancer [7]. Clinicians currently employ the American Joint Committee on Cancer (AJCC) staging system to predict patient prognosis and guide treatment [8]. The AJCC staging system, which assesses tumor stage based on anatomical information concerning the primary tumor (T), regional lymph nodes (N), and distant metastases (M), relies heavily on the absolute number of positive lymph nodes identified during surgery (pN stage). However, a fundamental limitation of this traditional approach is its strong dependence on the total number of lymph nodes harvested. When the total number of examined lymph nodes is insufficient, patients with a substantial burden of lymph node metastasis may be erroneously categorized into a lower risk tier. This phenomenon can lead to stage migration [9, 10] and an underestimation of mortality risk.

To address this inherent bias, the log odds of positive lymph nodes (LODDS) has been proposed as an alternative nodal evaluation metric [11]. Theoretically, LODDS mitigates this bias by simultaneously incorporating the counts of both positive and negative lymph nodes [12]. By including negative lymph nodes in the denominator, LODDS mathematically serves as a surrogate for the thoroughness of surgical clearance, thereby reflecting the true tumor burden more accurately than simple absolute counts. Although LODDS has demonstrated prognostic utility across various solid tumors, including breast, gastric, and colorectal cancers [13–15], its continuous prognostic value and incremental predictive utility within the highly heterogeneous YBC population remain insufficiently elucidated.

Previous prognostic modeling studies have frequently utilized conventional Cox proportional hazards models, though the potential impact of non-proportional hazards has not always been explicitly evaluated. Using a large population-based cohort from the Surveillance, Epidemiology, and End Results (SEER) database, the present study aimed to evaluate the prognostic value of LODDS for breast cancer-specific survival (BCSS) in YBC patients. As the objectives of etiologic association assessment and individualized risk prediction are not entirely identical, different modeling strategies were applied according to the specific analytic purpose. Stratified Cox proportional hazards models were used to estimate the independent association between LODDS and BCSS while accounting for major violations of the proportional hazards assumption. Separately, a multivariable prediction model incorporating continuous LODDS was developed using least absolute shrinkage and selection operator (LASSO)-based feature selection and a standard Cox regression framework, followed by bootstrap internal validation. We hypothesized that LODDS may provide incremental prognostic information beyond conventional nodal staging and could serve as a complementary indicator within existing prognostic assessment systems for YBC patients.

## Materials and methods

### Data source and study population

Data for this retrospective study were extracted from the SEER database using SEER*Stat software (version 9.0.42). We utilized the “Incidence - SEER Research Data, 17 Registries, Nov 2022 Sub (2000–2020)” dataset. Patients diagnosed with primary breast cancer between 2010 and 2015 were screened to ensure the availability of complete staging information based on the AJCC 7th edition. The inclusion criteria were defined as follows: (1) female patients aged 20–39 years at the time of diagnosis; (2) primary breast malignancy (International Classification of Diseases for Oncology, Third Edition [ICD-O-3] codes C50.0–C50.9) confirmed by positive histology; (3) the breast malignancy was the patient’s first and only primary tumor; (4) known marital status; and (5) complete follow-up and survival data available. Patients were subsequently excluded from the study based on the following criteria: (1) missing key clinicopathological variables; (2) a recorded survival time of exactly 0 months; (3) diagnosis identified solely via autopsy or death certificate; and (4) did not undergo primary breast surgery, as accurate pathological evaluation of lymph nodes is a prerequisite for reliable LODDS calculation. Following these rigorous criteria, the final enrolled cohort comprised 9,989 surgically treated patients, which was utilized in its entirety for model development and internal validation. The patient selection process is detailed in Figure 1. For missing data, a complete-case analysis approach was adopted, and patients with missing key clinicopathological variables were excluded from the study.

**Figure 1.**
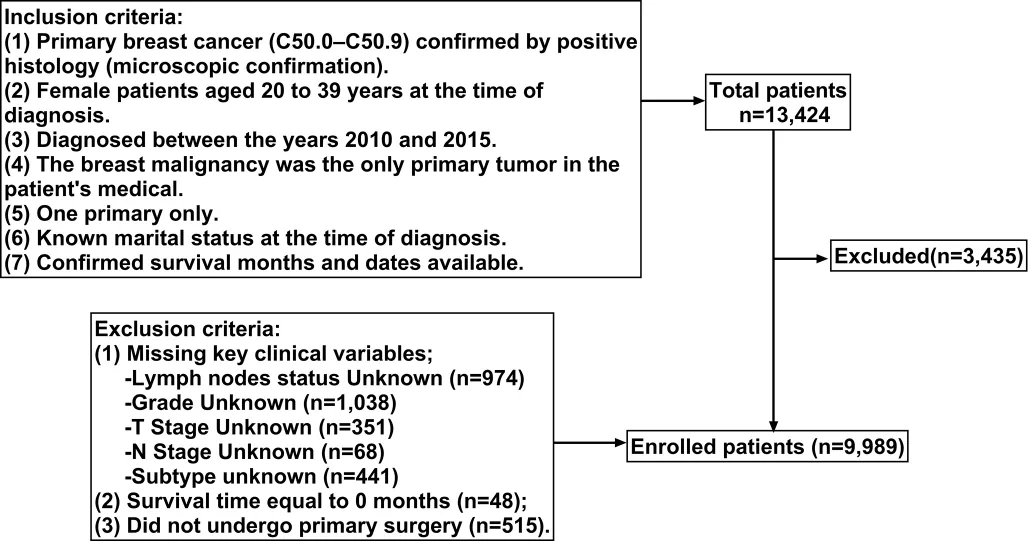
**Flowchart of patient selection.** Initial screening identified 13,424 female patients aged 20 to 39 years who were diagnosed with primary breast cancer between 2010 and 2015, in accordance with the established inclusion criteria. Following the exclusion of 3,435 patients, a total of 9,989 eligible patients were ultimately enrolled in the study. **Abbreviations:** n, number of patients; N, regional lymph node; T, primary tumor.

### Variable extraction and outcome definition

Candidate predictors extracted from the SEER database included demographic characteristics (age at diagnosis, race, and marital status), tumor-related factors (primary site, laterality, histological type, grade, T stage, N stage, M stage, examined lymph nodes, and positive lymph nodes), and treatment-related variables (surgery, radiotherapy, and chemotherapy). Subtype was defined based on hormone receptor (HR) and human epidermal growth factor receptor 2 (HER2) status. In the SEER database, the number of negative lymph nodes was deduced by subtracting positive nodes from total examined nodes. LODDS was then calculated using the empirical logistic transform: LODDS = log [(positive nodes + 0.5) / (negative nodes + 0.5)]. The 0.5 acts as a standard Haldane-Anscombe continuity correction to prevent mathematical singularities [11]. The primary endpoint of this study was BCSS, defined as the interval from the date of initial diagnosis to the date of death specifically caused by breast cancer.

### Ethical statement

This study utilized publicly available, de-identified data from the SEER database, which does not contain any personal identifiers. Therefore, this study was exempt from review and approval by the Institutional Review Board, and the requirement for informed consent was waived. All procedures performed in this study were in accordance with the ethical standards of the institutional and/or national research committee and with the Declaration of Helsinki.

### Statistical analysis

All statistical analyses were performed using R software (version 4.5.2). A two-sided *P*-value < 0.05 was considered statistically significant.

To ensure full methodological reproducibility, all clinicopathological and treatment variables were extracted and categorized using exact SEER database coding schemas (detailed in Table S1).

Demographic variables included age at diagnosis, race, and marital status. Age was analyzed as a categorical variable, stratified into four groups: 20–24, 25–29, 30–34, and 35–39 years. Race was classified as White, Black, and Other (which includes American Indian/Alaska Native and Asian/Pacific Islander). Marital status at the time of diagnosis was categorized as married and unmarried. Tumor-related characteristics encompassed the primary tumor site, laterality, histologic subtype, tumor grade, and standard AJCC staging. Primary tumor sites were categorized based on SEER primary site codes as the upper-inner quadrant (UIQ), lower-inner quadrant (LIQ), upper-outer quadrant (UOQ), lower-outer quadrant (LOQ), and others (including axillary tail, overlapping lesions, central portion, and breast, NOS). Histologic subtypes were identified using the International Classification of Diseases for Oncology, Third Edition (ICD-O-3) codes and were dichotomized into two categories: Infiltrating Ductal Carcinoma (IDC; 8500/3) and Other Invasive Carcinomas (comprising all other eligible breast malignancy codes). Tumor grade was classified as well-differentiated (Grade I), moderately differentiated (Grade II), and poorly differentiated (Grade III). Breast cancer molecular subtypes were defined into four distinct categories: HR-/HER2-, HR-/HER2+, HR+/HER2-, and HR+/HER2+. Treatment variables included surgical management, radiotherapy, and chemotherapy. Surgical treatment was categorized into mastectomy and breast-conserving surgery (BCS). The receipt of radiotherapy and chemotherapy was recorded as a binary variable (Yes or No). For the LASSO feature selection and multivariate Cox regression modeling, all candidate predictors were handled as categorical variables. They were entered into the models using dummy coding, with the lowest clinical risk category or the most epidemiologically frequent category prospectively designated as the baseline reference group.

**Feature selection:** To preserve the continuous prognostic information of LODDS and avoid potential data-driven dichotomization, LODDS was maintained as a continuous variable throughout all analyses. Prognostic feature selection utilized the LASSO regression model, with the optimal penalty parameter (λ) determined via 10-fold cross-validation according to the 1-standard-error criterion.

**Survival modeling strategy:** The proportional hazards (PH) assumption was assessed using scaled Schoenfeld residuals and corresponding time-dependent tests. Several covariates, particularly tumor grade and molecular subtype, exhibited deviations from the PH assumption (Table S2). To estimate the independent association between LODDS and BCSS, a multivariable stratified Cox proportional hazards model was constructed. This inferential model was stratified by tumor grade and molecular subtype, allowing for stratum-specific baseline hazard functions while retaining LODDS and other covariates as fixed effects. For nomogram development, an unstratified Cox proportional hazards model was employed to estimate individualized survival probabilities at fixed prediction horizons. Given the nomogram’s primary objective of risk prediction over etiologic effect estimation, model performance was evaluated predominantly through empirical measures of discrimination and calibration. Due to the cohort’s large sample size, some statistically significant departures from proportionality may exert limited influence on prediction performance at fixed clinical time horizons.

**Model performance and validation:** The nomogram’s predictive performance was evaluated across the entire cohort, with internal validation performed using a 1,000-resample bootstrap procedure for optimism correction. To mitigate potential overoptimism from model selection, the complete model-building process, including LASSO-based feature selection and penalty parameter tuning via cross-validation, was independently replicated within each bootstrap resample.

**Discrimination:** Discriminative ability was assessed using Harrell’s concordance index (C-index) and time-dependent receiver operating characteristic (ROC) analysis. Area under the curve (AUC) values were calculated at 3, 5, and 10 years. Continuous net reclassification improvement (NRI) and integrated discrimination improvement (IDI) were also calculated to evaluate the incremental prognostic contribution of continuous LODDS beyond the traditional tumor-node-metastasis (TNM) staging system.

**Table 1:** Baseline characteristics of patients

**Variables**	**Total (*n* ═ 9989)**
LODDS, Mean ± SD	−0.62 ± 0.52
Age, n(%)	
20–24	172 (1.72)
25–29	952 (9.53)
30–34	2847 (28.50)
35–39	6018 (60.25)
T stage, n (%)	
T1	3990 (39.94)
T2	4477 (44.82)
T3	1143 (11.44)
T4	379 (3.79)
N stage, n (%)	
N0	5132 (51.38)
N1	3463 (34.67)
N2	885 (8.86)
N3	509 (5.10)
M stage, n (%)	
M0	9682 (96.93)
M1	307 (3.07)
Race, n (%)	
Black	1406 (14.08)
White	7125 (71.33)
Others	1458 (14.60)
Primary site, n (%)	
LIQ	517 (5.18)
LOQ	861 (8.62)
UIQ	1258 (12.59)
UOQ	3338 (33.42)
Others	4015 (40.19)
Laterality, n (%)	
Left	5030 (50.36)
Right	4959 (49.64)
Grade, n (%)	
Grade I	835 (8.36)
Grade II	3488 (34.92)
Grade III	5666 (56.72)
ICD-O-3, n (%)	
IDC	8473 (84.82)
Other invasive carcinomas	1516 (15.18)
Surgery, n (%)	
BCS	2908 (29.11)
Mastectomy	7081 (70.89)
Radiotherapy, n (%)	
No	4944 (49.49)
Yes	5045 (50.51)
Chemotherapy, n (%)	
No	1874 (18.76)
Yes	8115 (81.24)
Subtype, n (%)	
HR–/HER2–	1935 (19.37)
HR–/HER2+	675 (6.76)
HR+/HER2–	5573 (55.79)
HR+/HER2+	1806 (18.08)
Year at diagnose, n (%)	
2010	1590 (15.92)
2011	1603 (16.05)
2012	1664 (16.66)
2013	1644 (16.46)
2014	1673 (16.75)
2015	1815 (18.17)
Marital status, n (%)	
Married	6155 (61.62)
Unmarried	3834 (38.38)

**Abbreviations:** BCS, breast-conserving surgery; HER2, human epidermal growth factor receptor 2; HR, hormone receptor; ICD-O-3, International Classification of Diseases for Oncology, Third Edition; IDC, invasive ductal carcinoma; LIQ, lower-inner quadrant; LODDS, log odds of positive lymph nodes; LOQ, lower-outer quadrant; M, distant metastasis; N, regional lymph node; SD, standard deviation; T, primary tumor; UIQ, upper-inner quadrant; UOQ, upper-outer quadrant.

**Calibration:** Calibration was assessed both visually and quantitatively. Bootstrap-corrected calibration curves were plotted at 3, 5, and 10 years. Quantitative calibration was evaluated using the Brier score (reflecting overall accuracy) alongside the mean absolute error (*Eavg*) and maximum absolute error (*Emax*).

**Clinical utility:** Decision curve analysis (DCA) was performed to evaluate the potential clinical net benefit of the nomogram across a range of clinically relevant threshold probabilities at 3, 5, and 10 years.

The reporting of this predictive model adheres to the updated Transparent Reporting of a multivariable prediction model for Individual Prognosis Or Diagnosis + Artificial Intelligence (TRIPOD+AI) guidelines, ensuring methodological transparency [16].

## Results

### Baseline characteristics of the study population

Initially, 13,424 young female patients diagnosed with primary breast cancer were identified from the SEER database. After applying exclusion criteria, a final cohort of 9,989 patients was enrolled. The patient selection process is detailed in Figure 1. The mean follow-up time for the entire cohort was 82.84 months (standard deviation [SD] ═ 30.03), during which 1,361 (13.62%) breast cancer-specific deaths were observed. Baseline demographic and clinicopathological characteristics are summarized in Table 1.

### Feature selection via LASSO regression

To identify prognostic features and mitigate potential overfitting, the LASSO Cox regression model was applied to the entire cohort. Following 10-fold cross-validation, the optimal penalty parameter (λ) was selected based on the 1-standard error of the minimum criteria (Figure 2). The LASSO model successfully reduced data dimensionality, retaining 6 non-zero coefficient variables. Notably, continuous LODDS was consistently selected as a key feature (Coefficient = 0.467), even when the traditional N stage (N1, N2, N3) was included in the model. This finding suggests that LODDS provides incremental prognostic value beyond a mere count of positive lymph nodes.

**Figure 2.**
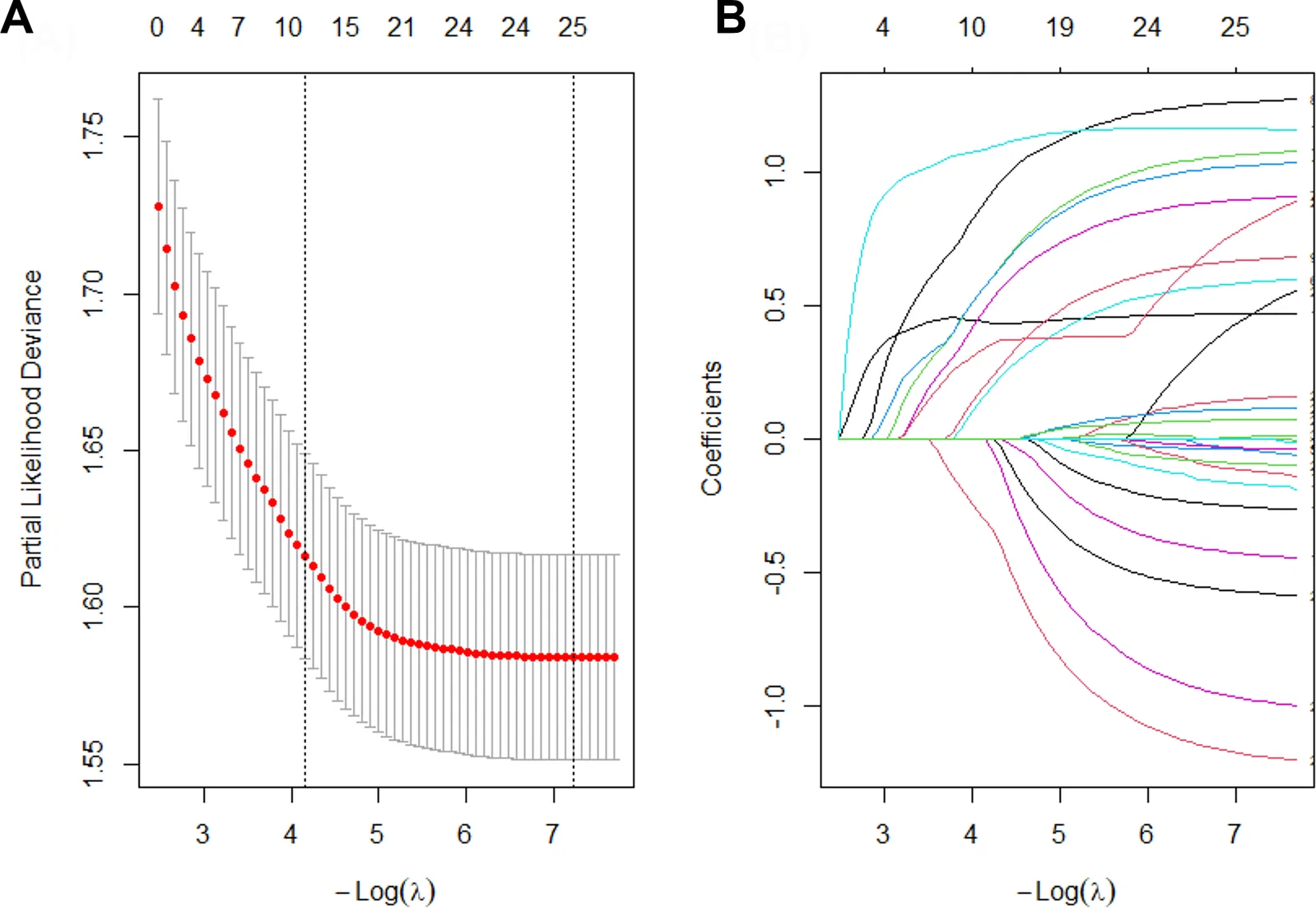
**LASSO-based selection of prognostic variables for the nomogram.** (A) Ten-fold cross-validation curve of the LASSO Cox regression model. Partial likelihood deviance is shown according to −log(λ), with red points representing the mean cross-validated deviance and gray error bars indicating standard errors. The vertical dotted lines mark the λ values corresponding to the minimum cross-validated error and the 1-standard-error criterion, which was used to select a more parsimonious prognostic model. The upper axis indicates the number of non-zero coefficients retained at each λ value. (B) Coefficient profiles of candidate predictors across different λ values. As penalization changes, regression coefficients are progressively shrunk toward zero, allowing selection of the most informative prognostic variables. Continuous LODDS was retained in the selected model, supporting its incremental prognostic value beyond conventional nodal staging. **Abbreviations**: LASSO, least absolute shrinkage and selection operator; LODDS, log odds of positive lymph nodes; λ, lambda regularization parameter.

### Independent prognostic value of LODDS

To evaluate the independent prognostic value of LODDS while accounting for deviations from the PH assumption, a multivariable stratified Cox regression analysis was performed (Figure 3). Schoenfeld residual testing indicated non-proportional hazards for several covariates, with the strongest deviations observed for tumor grade and molecular subtype. Consequently, these variables were incorporated as stratification factors in the inferential model. After adjustment for standard clinicopathological covariates, continuous LODDS remained a significant independent predictor of worse BCSS (HR = 1.60, 95% confidence interval [CI]: 1.45–1.77, *P <* 0.001). Advanced T stage, N stage, and the presence of distant metastasis (M1) were also independently associated with increased breast cancer-specific mortality (all *P <* 0.001). Furthermore, variance inflation factor (VIF) analysis confirmed the absence of severe multicollinearity among covariates, particularly between continuous LODDS (VIF = 1.58) and N stage (VIF = 1.99).

**Figure 3.**
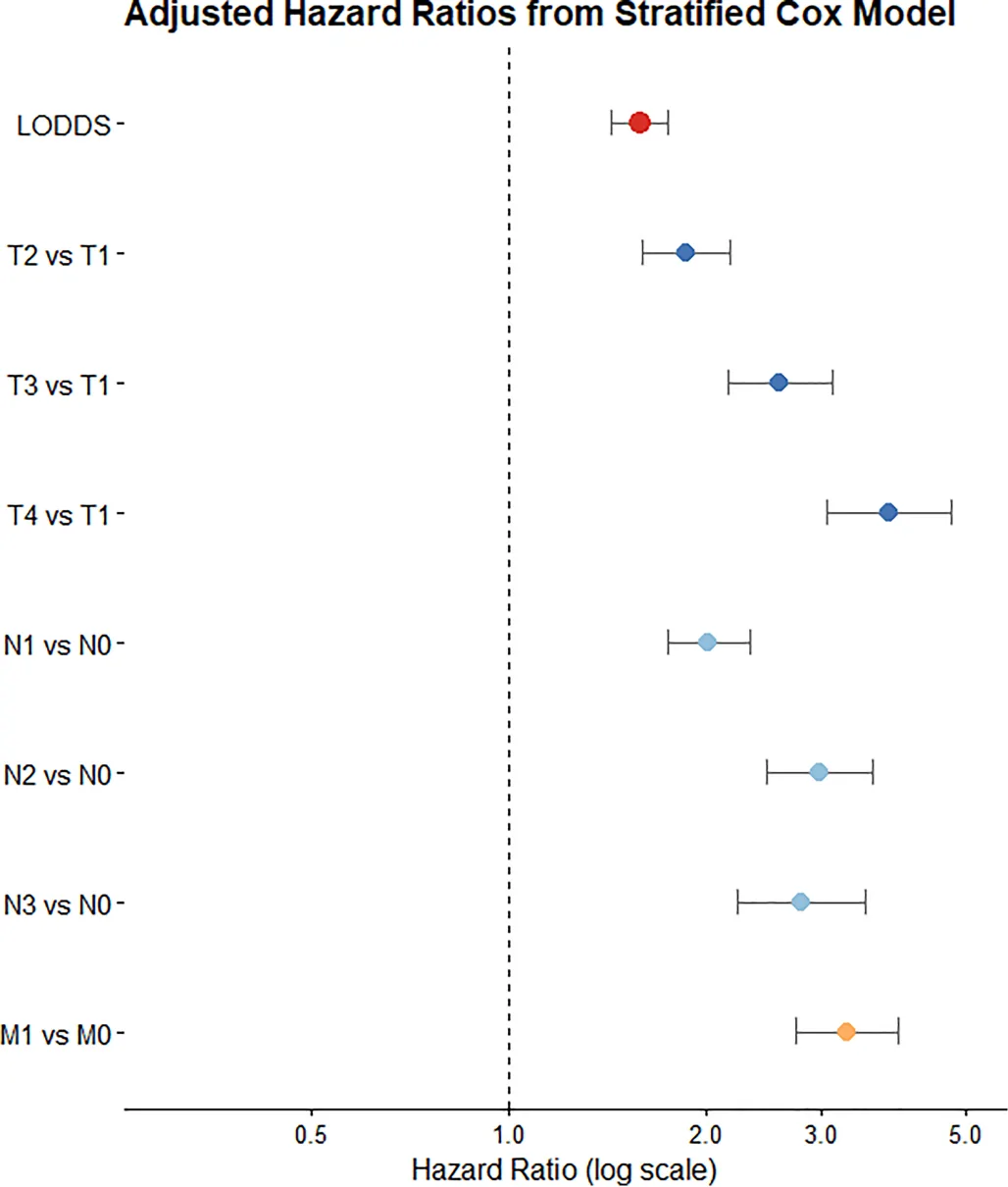
**Adjusted hazard ratios for breast cancer-specific survival from the stratified Cox model.** Forest plot showing adjusted hazard ratios and 95% confidence intervals for continuous LODDS and TNM stage variables. The model was stratified by tumor grade and molecular subtype to account for non-proportional hazards. The vertical dashed line at HR = 1.0 represents no association with breast cancer-specific mortality. Estimates to the right of the reference line indicate increased mortality risk compared with the corresponding reference categories (T1, N0, and M0). Continuous LODDS and more advanced T, N, and M stages were associated with poorer BCSS. **Abbreviations**: BCSS, breast cancer-specific survival; CI, confidence interval; HR, hazard ratio; LODDS, log odds of positive lymph nodes; M, distant metastasis; N, regional lymph node; T, primary tumor; TNM, tumor-node-metastasis.

**Figure 4.**
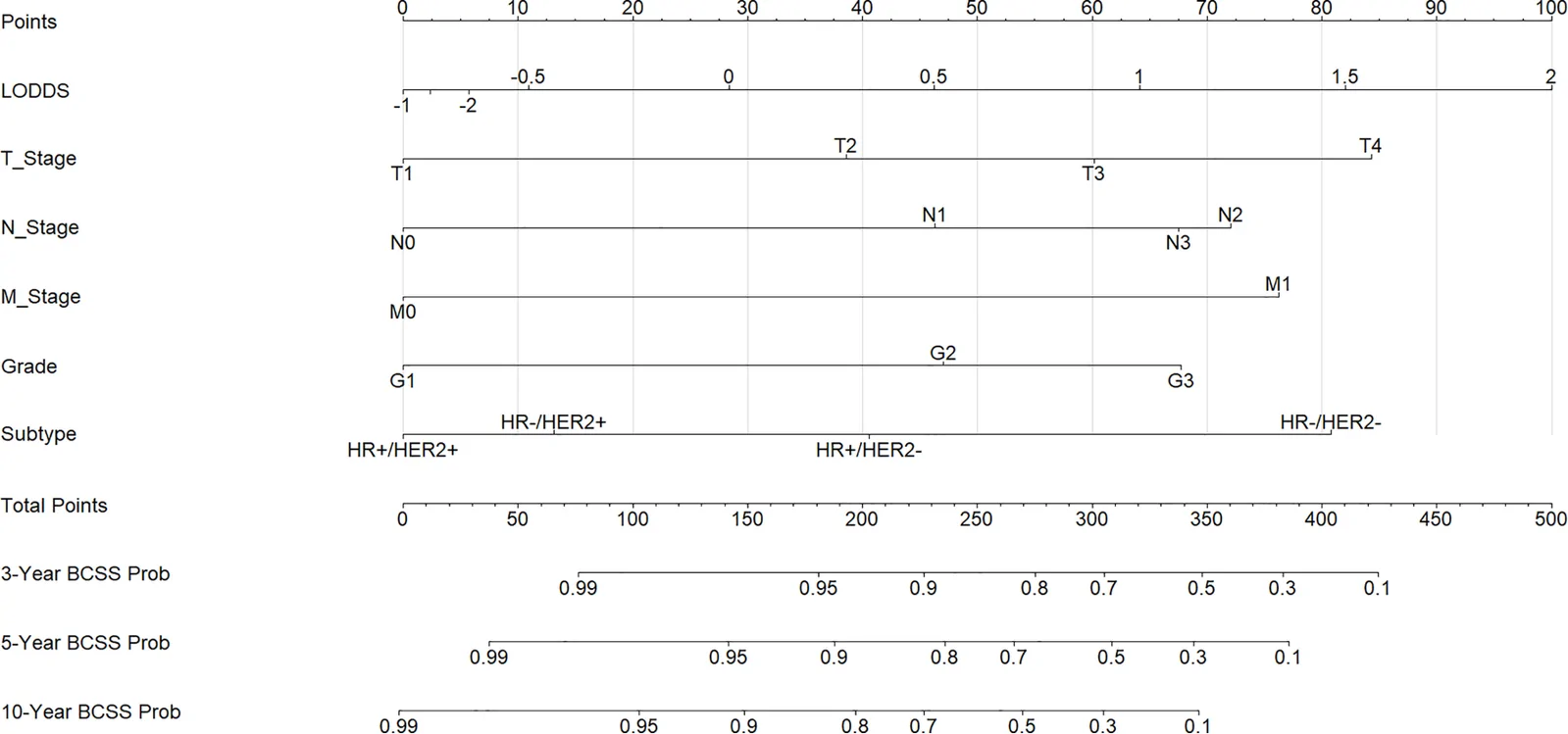
**Nomogram for individualized prediction of 3-, 5-, and 10-year breast cancer-specific survival.** The nomogram was constructed using continuous LODDS and selected clinicopathological predictors, including T stage, N stage, M stage, histological grade, and molecular subtype. For each patient, points are assigned by locating the corresponding value or category on each predictor axis and projecting vertically to the “Points” scale. The individual scores are then summed to obtain the “Total Points,” which are projected downward to estimate the predicted 3-, 5-, and 10-year BCSS probabilities. Higher total points indicate a lower predicted probability of BCSS. **Abbreviations**: BCSS, breast cancer-specific survival; G, histological grade; HER2, human epidermal growth factor receptor 2; HR, hormone receptor; LODDS, log odds of positive lymph nodes; M, distant metastasis; N, regional lymph node; T, primary tumor.

### Model performance, nomogram construction and internal validation

For practical clinical application, an unstratified standard Cox model, incorporating continuous LODDS and other LASSO-selected clinicopathological variables, was developed using the entire cohort to construct a prognostic nomogram (Figure 4). To account for potential overoptimism associated with model development and feature selection, the model’s performance was assessed using a 1,000-resample bootstrap optimism-correction procedure. The apparent C-index of the nomogram was 0.7808, which remained stable at 0.7766 after bootstrap correction. This demonstrated superior discriminative performance compared to the traditional TNM staging model (C-index = 0.736). Time-dependent ROC analysis further corroborated this sustained accuracy. The nomogram’s time-dependent AUCs reached 0.843, 0.810, and 0.777 at 3, 5, and 10 years, respectively. These results consistently outperformed both the traditional TNM staging model (AUCs = 0.784, 0.767, and 0.752) and the LODDS alternative model across all evaluated time points (Figure 5).

**Figure 5.**
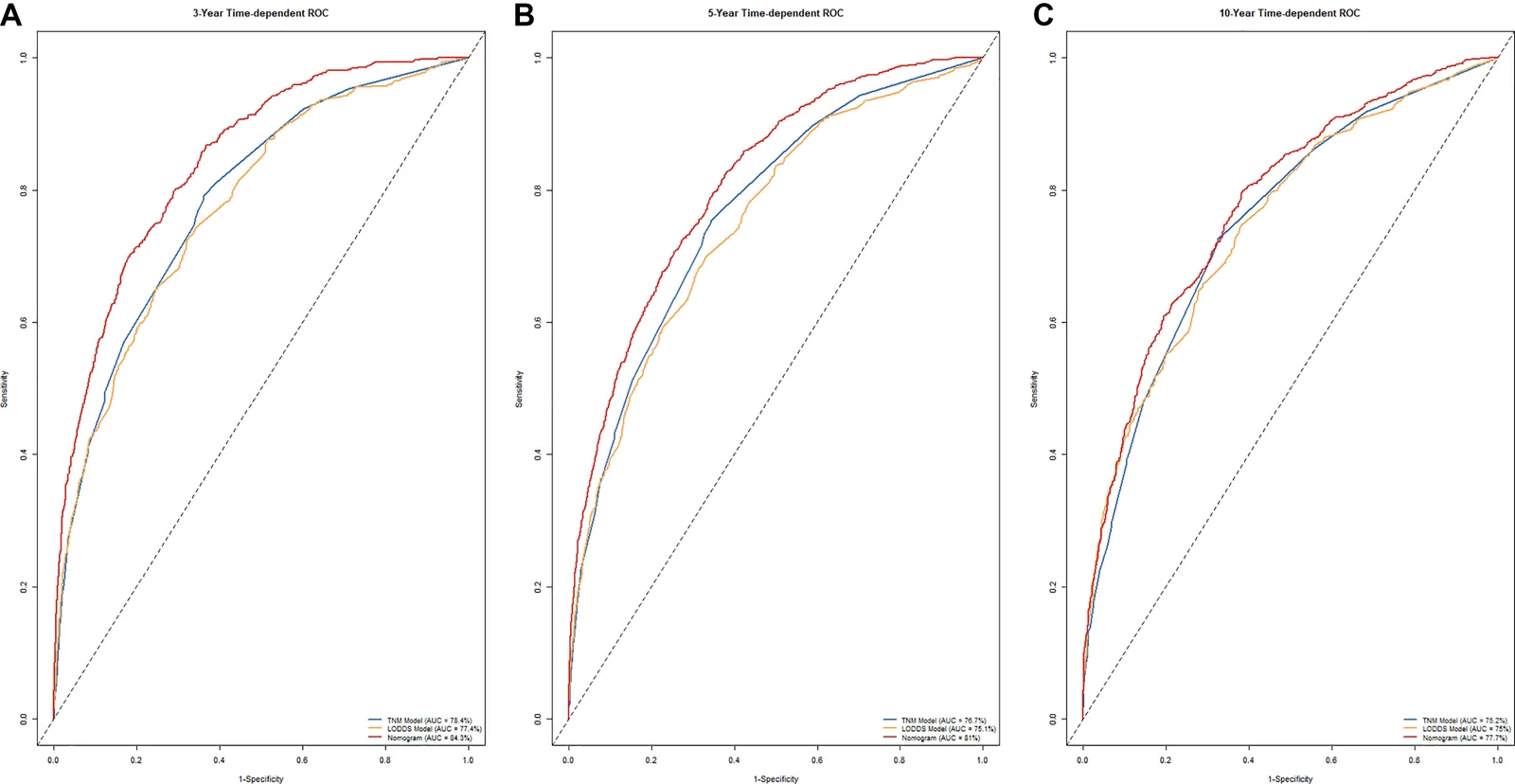
**Time-dependent ROC curves for predicting 3-, 5-, and 10-year breast cancer-specific survival.** Time-dependent ROC curves compare the discriminative performance of the traditional TNM staging model, the LODDS alternative model, and the LODDS-integrated nomogram in the entire study cohort. (A) ROC curves for 3-year BCSS prediction. (B) ROC curves for 5-year BCSS prediction. (C) ROC curves for 10-year BCSS prediction. The corresponding AUC values are displayed within each panel. The nomogram showed the highest discriminatory ability at all evaluated time points, with AUCs of 0.843, 0.810, and 0.777 at 3, 5, and 10 years, respectively, outperforming both the TNM model and the LODDS alternative model. The dashed diagonal line represents the reference line for a non-informative model. **Abbreviations:** AUC, area under the curve; BCSS, breast cancer-specific survival; LODDS, log odds of positive lymph nodes; ROC, receiver operating characteristic; TNM, tumor-node-metastasis.

**Figure 6.**
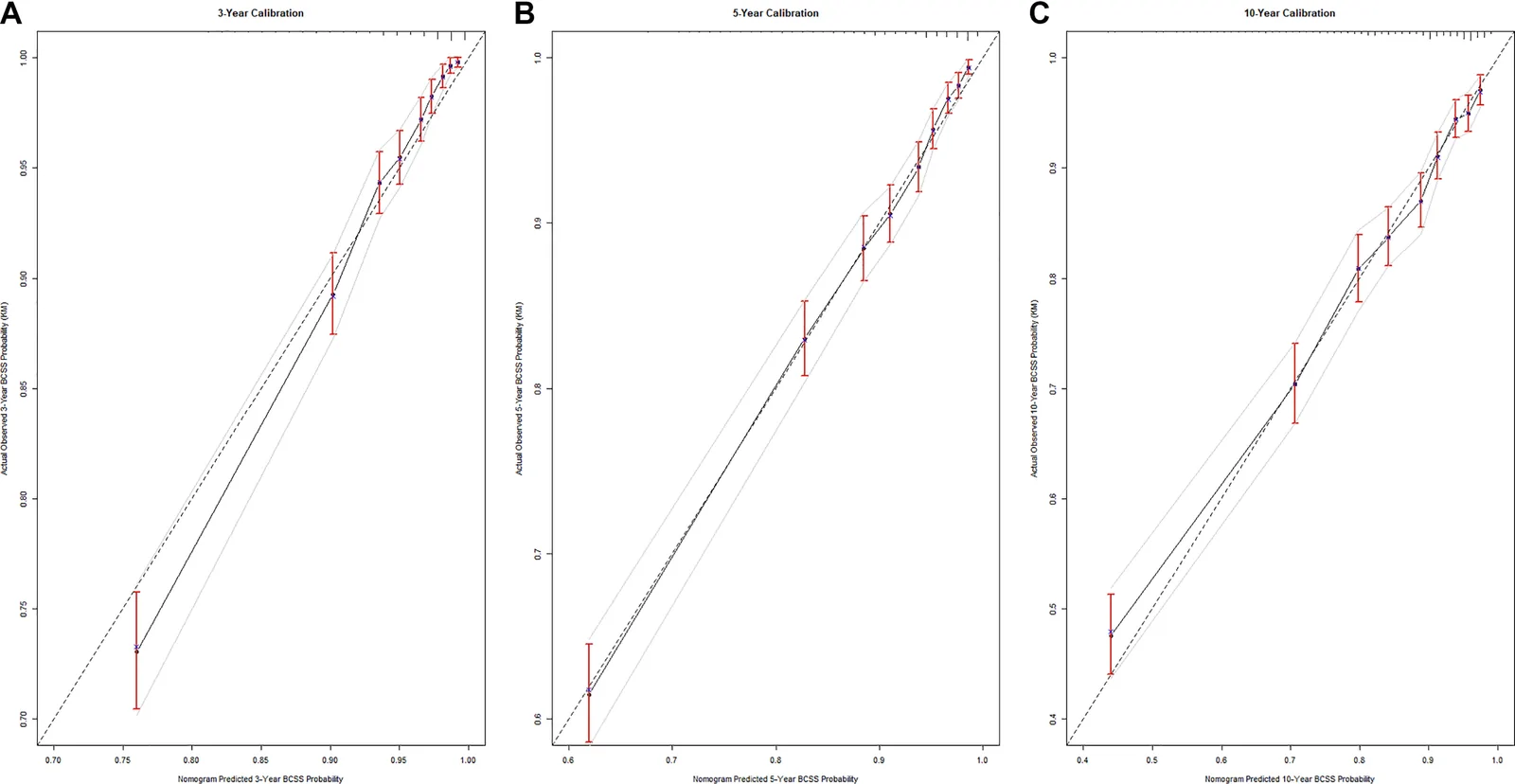
**Calibration curves of the prognostic nomogram for predicting BCSS.** The calibration curves assess the concordance between the nomogram-predicted survival probabilities (x-axis) and the actual observed survival probabilities (y-axis) at 3, 5, and 10 years. The 45-degree dashed line represents an ideal predictive model, wherein the predicted probabilities precisely align with the actual observed probabilities. (A) Calibration curves for 3-year BCSS in the entire cohort. (B) Calibration curves for 5-year BCSS in the entire cohort. (C) Calibration curves for 10-year BCSS in the entire cohort. The close alignment of the solid curves with the dashed reference line indicates that the nomogram demonstrates high calibration accuracy across the study cohort. **Abbreviation**: BCSS, breast cancer-specific survival.

**Figure 7.**
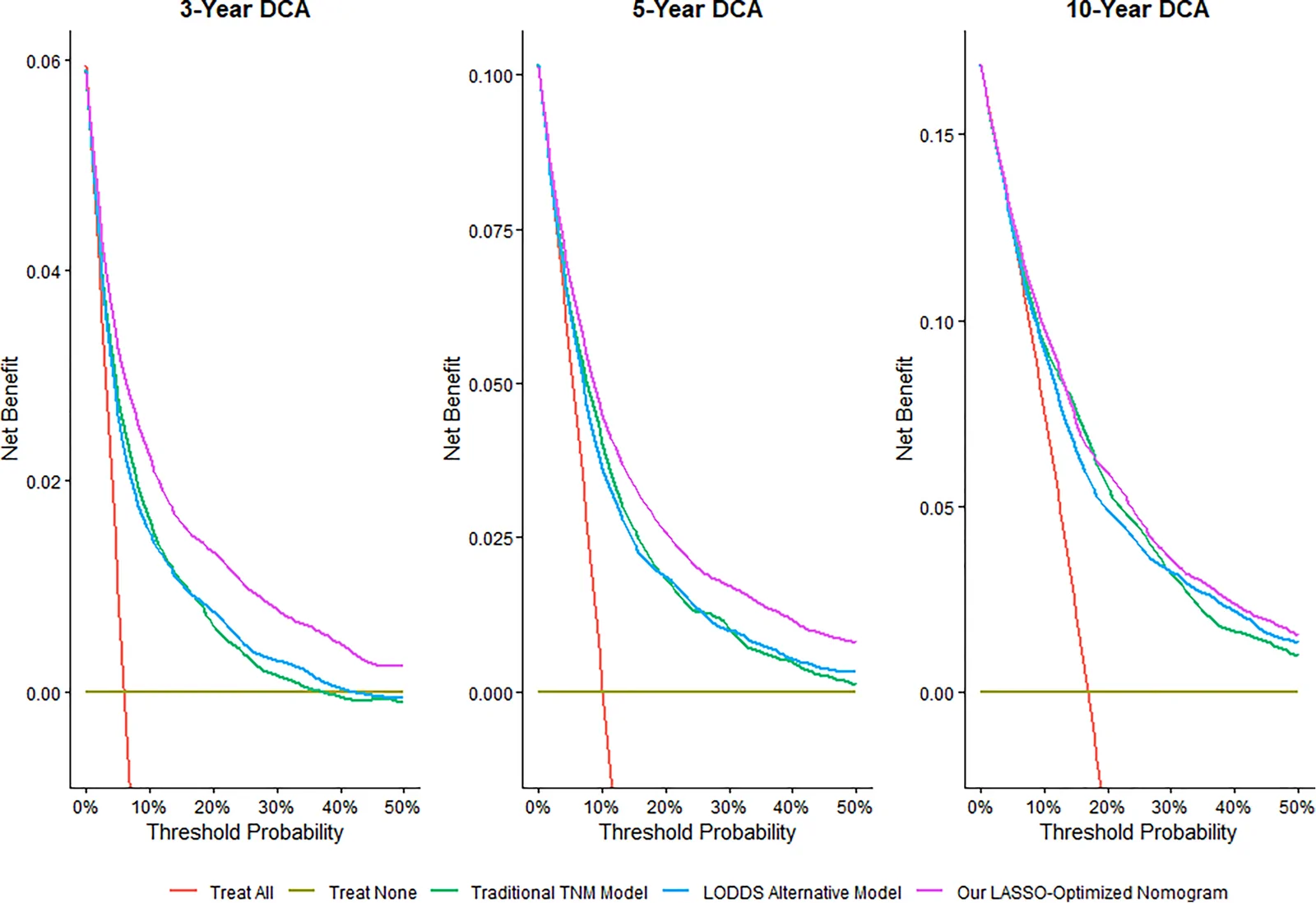
**Decision curve analysis of the prediction models for 3-, 5-, and 10-year breast cancer-specific survival.** Decision curve analysis was performed to compare the clinical utility of the traditional TNM staging model, the LODDS alternative model, and the LASSO-optimized nomogram incorporating continuous LODDS and selected clinicopathological predictors. The left, middle, and right panels show net benefit across threshold probabilities for 3-, 5-, and 10-year BCSS prediction, respectively. The x-axis represents the threshold probability, and the y-axis represents net benefit. The “Treat None” line represents a strategy in which no patients are classified as high risk, whereas the “Treat All” line represents a strategy in which all patients are classified as high risk. Across a broad range of clinically relevant threshold probabilities, the LASSO-optimized nomogram showed higher net benefit than the TNM and LODDS alternative models, supporting its potential value for individualized risk stratification in young breast cancer patients. **Abbreviations**: BCSS, breast cancer-specific survival; DCA, decision curve analysis; LASSO, least absolute shrinkage and selection operator; LODDS, log odds of positive lymph nodes; TNM, tumor-node-metastasis.

Beyond baseline discrimination, the incremental predictive value of integrating continuous LODDS into the traditional TNM system was quantified using continuous NRI and IDI analyses. The addition of continuous LODDS significantly improved risk reclassification for BCSS across all evaluated time points. Specifically, at 5 years, the nomogram yielded a continuous NRI of 0.278 (95% CI: 0.228–0.304, *P <* 0.001) and an IDI of 0.034 (95% CI: 0.026–0.044, *P <* 0.001). Similar improvements were consistently observed at the 3-year (NRI = 0.332, *P <* 0.001; IDI = 0.033, *P <* 0.001) and 10-year intervals (NRI = 0.210, *P <* 0.001; IDI = 0.032, *P*< 0.001). This suggests that the continuous LODDS-integrated model correctly reclassifies a significant proportion of YBC patients whose mortality risks were misjudged by the TNM system alone.

Calibration was assessed both visually and quantitatively. The bootstrap-corrected calibration curves at 3, 5, and 10 years demonstrated good agreement, closely adhering to the ideal 45-degree reference line (Figure 6). Quantitatively, the model exhibited high accuracy across all time points. Specifically, the model achieved a 3-year Brier score of 0.0469 (*Eavg* ═ 0.0098, *Emax* ═ 0.0137), a 5-year Brier score of 0.0757 (*Eavg* ═ 0.0047, *Emax* ═ 0.0081), and a 10-year Brier score of 0.1151 (*Eavg* ═ 0.0107, *Emax* ═ 0.0232). These findings suggest acceptable agreement between predicted and observed outcomes across the evaluated time horizons.

Finally, DCA was performed to quantify the clinical net benefit. The DCA curves confirmed that within a wide range of clinically reasonable threshold probabilities, employing the continuous LODDS-integrated nomogram consistently provided a higher clinical net benefit at 3, 5, and 10 years compared to “treat-all,” “treat-none,” or traditional TNM staging strategies (Figure 7).

## Discussion

Lymph node status is a critical prognostic factor in both early and locally advanced breast cancer. This large population-based study, encompassing 9,989 YBC patients, investigated the prognostic significance of the LODDS in relation to BCSS. Continuous LODDS demonstrated an independent association with BCSS after multivariable adjustment, offering additional prognostic insights beyond traditional nodal staging metrics.

The clinical relevance of this research is underscored by the unique epidemiological characteristics of YBC. While women under 40 constitute a relatively small proportion of breast cancer cases in Western populations, East Asian populations, particularly China, report significantly higher proportions. Furthermore, the median age at diagnosis in China is younger than that observed in Western countries [17]. These demographic disparities highlight the importance of refined prognostic stratification in younger patient cohorts.

One notable advantage of LODDS is its reduced susceptibility to the stage migration phenomenon. The current AJCC TNM nodal classification, primarily based on the absolute number of positive lymph nodes, can be influenced by the total number of examined lymph nodes. In cases of limited nodal evaluation, patients with substantial nodal involvement may be erroneously assigned to lower pN categories, leading to an underestimation of prognosis.

This issue is particularly pertinent in YBC, a population characterized by more aggressive clinicopathological features, including higher histological grade, increased proliferative activity, and a greater prevalence of triple-negative disease [18]. By integrating information from both positive and negative lymph nodes, LODDS partially accounts for the extent of nodal evaluation, potentially providing a more stable representation of nodal burden than positive-node counts alone.

An additional observation from the variable selection process was the retention of both conventional N stage and continuous LODDS in the final LASSO-selected model. Given the related yet distinct information captured by these variables, this finding suggests that LODDS may offer prognostic information that complements, rather than merely replaces, conventional nodal staging. While N stage primarily reflects the absolute extent of nodal metastasis, LODDS additionally incorporates the balance between positive and negative lymph nodes. This distinction may partially explain why both variables contributed to the final prediction model despite their biological correlation. Previous studies evaluating the prognostic role of LODDS in YBC populations remain relatively limited [19, 20]. More broadly, our findings align with recent efforts to enhance prognostic assessment in breast cancer through quantitative risk modeling approaches using extensive population-based datasets [21, 22].

To facilitate individualized risk estimation, we developed a prognostic nomogram incorporating continuous LODDS alongside conventional clinicopathological variables. The final LASSO-selected model exhibited strong discriminative performance, evidenced by a bootstrap-corrected C-index of 0.781 and a 5-year AUC of 0.810. In this study, LODDS was maintained as a continuous variable throughout model development. Prior methodological research has demonstrated that data-driven dichotomization of continuous predictors can lead to information loss, reduced statistical efficiency, and optimistic estimation of prognostic effects [23, 24]. Retaining LODDS in its continuous form preserved its prognostic gradient and avoided arbitrary cutoff selection. Decision curve analysis further indicated that the LODDS-integrated model provided a higher estimated net benefit across a range of clinically relevant threshold probabilities compared to the conventional staging-based approach.

Several limitations of this study warrant consideration when interpreting the findings. First, the retrospective design of the SEER database and the use of a complete-case analysis approach may introduce selection bias, potentially affecting the generalizability of the results. Second, the SEER database lacks detailed information regarding systemic therapies and molecular characteristics. Data on specific chemotherapy regimens, targeted therapies, endocrine therapy adherence, Ki-67 proliferation index, breast cancer susceptibility gene (BRCA) mutation status, and other genomic markers were unavailable. Given the biological heterogeneity of YBC, the omission of these variables may have resulted in residual confounding. Third, although internal validation using a 1,000-resample bootstrap procedure demonstrated acceptable model performance, external validation was not performed in the current study. Additionally, several variables exhibited evidence of non-proportional hazards, which may influence long-term risk estimation despite the modeling strategies applied. Future studies incorporating more comprehensive treatment and molecular data, along with independent external validation cohorts, are necessary before broader clinical implementation of this model can be considered.

## Conclusion

Continuous LODDS was independently associated with BCSS in YBC patients and provided additional prognostic information beyond conventional nodal staging variables. The internally validated prediction model, incorporating continuous LODDS, demonstrated good discrimination and calibration within the SEER cohort. These findings suggest that LODDS may serve as a complementary prognostic indicator for risk stratification in YBC; however, further external validation is required before broader clinical application.

## Supplemental data

**Table S1:** SEER variables and codes used for cohort selection and predictor definition

**Variable category**	SEER*Stat variable name	**Categories/codes included in the study**
Eligibility criteria (cohort selection)		
Site and morphology	Site recode ICD-O-3 2023 Revision	Breast
	Primary site - labeled	C50.0–C50.9 (All breast sub-sites)
	Diagnostic confirmation	Microscopically confirmed
Age	Age recode with <1 year olds	20–24 years, 25–29 years, 30-34 years, 35–39 years
Sex	Sex	Female
Year of diagnosis	Year of diagnosis	2010, 2011, 2012, 2013, 2014, 2015
Primary status	Sequence number	One primary only
Survival data	Survival months flag	Complete dates are available and there are more than 0 days of survival
Marital status	Marital status at diagnosis	Excluded “Unknown”
Main predictors (clinicopathological features)		
T stage	Derived AJCC T, 7th ed (2010-2015)	T1, T2, T3, T4 (Excluded TX, T0)
N stage	Derived AJCC N, 7th ed (2010-2015)	N0, N1, N2, N3 (Excluded NX)
M stage	Derived AJCC M, 7th ed (2010-2015)	M0, M1 (Excluded MX)
Tumor grade	Grade (thru 2017)	Grade I, II, III /IV (Excluded Unknown)
Breast subtype	Breast Subtype (2010+)	HR–/HER2–, HR–/HER2+, HR+/HER2–, HR+/HER2+ (Excluded unknown)
Surgery	RX Summ–Surg Prim Site (1998+)	Breast conserving surgery (Codes 20-24); Mastectomy (Codes 30–80) (Excluded no surgery/Unknown)
Lymph node evaluation	Regional nodes examined (1988+)	Exact number (excluded unknown). Patients with zero examined lymph nodes were retained if complete nodal information was available. No minimum nodal-evaluation threshold was imposed
	Regional nodes positive (1988+)	Exact number (excluded unknown)

**Abbreviations:** AJCC, American Joint Committee on Cancer; HER2, human epidermal growth factor receptor 2; HR, hormone receptor; ICD-O-3, International Classification of Diseases for Oncology, Third Edition; M, distant metastasis; N, regional lymph node; RX Summ, treatment summary; SEER, Surveillance, Epidemiology, and End Results; T, primary tumor.

**Table S2:** Schoenfeld residual tests for the proportional hazards assumption in the multivariable Cox regression model

**Variable**	**Chi-square**	**df**	***P* value**
LODDS	0.553	1	0.457
T stage	20.575	3	0.00013
N stage	17.156	3	0.00066
M stage	2.339	1	0.126
Grade	89.008	2	<2e-16
Subtype	212.081	3	<2e-16
Global	268.632	13	<0.001

The proportional hazards assumption was evaluated using scaled Schoenfeld residuals and associated time-dependent tests. A two-sided *P* value of less than 0.05 was deemed indicative of a deviation from the proportional hazards assumption. Due to the cohort’s large sample size, even modest deviations from proportionality may achieve statistical significance, despite having minimal practical implications for predictive performance. **Abbreviations**: Df, degrees of freedom; LODDS, log odds of positive lymph nodes; M, distant metastasis; N, regional lymph node; T, primary tumor.

## Data Availability

The datasets analyzed during the current study are available in the Surveillance, Epidemiology, and End Results (SEER) Program database (www.seer.cancer.gov). Access to these datasets requires the submission of a research proposal and the signing of the SEER data-use agreement. The processed data supporting the conclusions of this article are available from the corresponding author upon reasonable request.

## References

[1] Bray F, Laversanne M, Sung H (2024). Global cancer statistics 2022:GLOBOCAN estimates of incidence and mortality worldwide for 36 cancers in 185 countries. CA Cancer J Clin.

[2] Wang H, Tsai Y, Dong Y, Liu JJ (2022). Young adult cancer incidence trends in Taiwan and the U.S. from 2002 to 2016. Cancer Epidemiol.

[3] Lima SM, Kehm RD, Swett K, Gonsalves L, Terry MB (2020). Trends in parity and breast cancer incidence in US women younger than 40 years from 1935 to 2015. JAMA Netw Open.

[4] Paluch-Shimon S, Cardoso F, Partridge AH (2020). ESO-ESMO 4th international consensus guidelines for breast cancer in young women (BCY4). Ann Oncol.

[5] Anastasiadi Z, Lianos GD, Ignatiadou E, Harissis HV, Mitsis M (2017). Breast cancer in young women: an overview. Updates Surg.

[6] Yang Y, Wei W, Jin L (2022). Comparison of the characteristics and prognosis between very young women and older women with breast cancer: a multi-institutional report from China. Front Oncol.

[7] Weiser R, Polychronopoulou E, Haque W (2021). Prognosis and chemotherapy use in breast cancer patients with multiple lymphatic micrometastases: an NCDB analysis. Ann Surg Oncol.

[8] Teichgraeber DC, Guirguis MS, Whitman GJ (2021). Breast cancer staging: updates in the AJCC cancer staging manual, 8th edition, and current challenges for radiologists, from the AJR special series on cancer staging. AJR Am J Roentgenol.

[9] Shaha AR, Migliacci JC, Nixon IJ (2019). Stage migration with the new American Joint Committee on Cancer (AJCC) staging system (8th edition) for differentiated thyroid cancer. Surgery.

[10] Sormani MP (2009). The Will Rogers phenomenon: the effect of different diagnostic criteria. J Neurol Sci.

[11] Vinh-Hung V, Verschraegen C, Promish DI (2004). Ratios of involved nodes in early breast cancer. Breast Cancer Res.

[12] Sun Z, Xu Y, Li DM (2010). Log odds of positive lymph nodes: a novel prognostic indicator superior to the number-based and the ratio-based N category for gastric cancer patients with R0 resection. Cancer.

[13] Scarinci A, Di Cesare T, Cavaniglia D (2018). The impact of log odds of positive lymph nodes (LODDS) in colon and rectal cancer patient stratification: a single-center analysis of 323 patients. Updates Surg.

[14] Wang C, Fang Y, Zhang Y (2025). A LODDS-based nomogram for overall and cancer-specific survival in stage III–IV gastric signet ring cell carcinoma. Front Mol Biosci.

[15] Güner E, Sarıdemir S, Özaslan C (2026). Prognostic value of metastatic lymph-node ratio and log-odds of positive nodes after neoadjuvant chemotherapy in breast cancer. World J Surg Oncol.

[16] Collins GS, Moons KGM, Dhiman P (2024). TRIPOD+AI statement: updated guidance for reporting clinical prediction models that use regression or machine learning methods. BMJ.

[17] Yap YS, Lu YS, Tamura K (2019). Insights into breast cancer in the East vs the West: a review. JAMA Oncol.

[18] Zhu JW, Charkhchi P, Adekunte S, Akbari MR (2023). What is known about breast cancer in young women?. Cancers (Basel).

[19] Liu R, Xiao Z, Hu D (2021). Cancer-specific survival outcome in early-stage young breast cancer: evidence from the SEER database analysis. Front Endocrinol (Lausanne).

[20] Li Y, Tao X, Ye Y (2024). Prognostic nomograms for young breast cancer: a retrospective study based on the SEER and METABRIC databases. Cancer Innov.

[21] Panda NR, Mahanta KL, Pati JK (2024). Development of prognostic model and multivariate analysis for breast cancer survival patients using SEER database. J Assoc Med Sci.

[22] Panda NR, Rajalakshmi R, Pramanik S, Donganont M, Raut PK (2025). Advancing breast cancer diagnosis: a comprehensive machine learning approach for predicting malignant and benign cases with precision and insight in a neutrosophic environment using neutrosophic numbers. Neutrosophic Sets Syst.

[23] Ewald B (2006). Post hoc choice of cut points introduced bias to diagnostic research. J Clin Epidemiol.

[24] Bhandari PM, Levis B, Neupane D (2021). Data-driven methods distort optimal cutoffs and accuracy estimates of depression screening tools: a simulation study using individual participant data. J Clin Epidemiol.

